# SREBP-1 Has a Prognostic Role and Contributes to Invasion and Metastasis in Human Hepatocellular Carcinoma

**DOI:** 10.3390/ijms15057124

**Published:** 2014-04-25

**Authors:** Chao Li, Wei Yang, Junli Zhang, Xin Zheng, Yingmin Yao, Kangsheng Tu, Qingguang Liu

**Affiliations:** Department of Hepatobiliary Surgery, the First Affiliated Hospital of Xi’an Jiaotong University, Xi’an 710061, China; E-Mails: lixiaomao1989@126.com (C.L.); drbobyang@163.com (W.Y.); xjtuzjl@stu.xjtu.edu.cn (J.Z.); xin.zheng.xjtu@gmail.com (X.Z.); yaoyingmin@sina.com (Y.Y.)

**Keywords:** SREBP-1, hepatocellular carcinoma, biomarker, prognosis, invasion, metastasis

## Abstract

Sterol regulatory element-binding protein 1 (SREBP-1) is a well-known nuclear transcription factor involved in lipid synthesis. Recent studies have focused on its functions in tumor cell proliferation and apoptosis, but its role in cell migration and invasion, especially in hepatocellular carcinoma (HCC), is still unclear. In this study, we found that the expression of SREBP-1 in HCC tissues was significantly higher than those in matched tumor-adjacent tissues (*p* < 0.05). SREBP-1 was expressed at significantly higher levels in patients with large tumor size, high histological grade and advanced tumor-node-metastasis (TNM) stage (*p* < 0.05). The positive expression of SREBP-1 correlated with a worse 3-year overall and disease-free survival of HCC patients (*p* < 0.05). Additionally, SREBP-1 was an independent factor for predicting both 3-year overall and disease-free survival of HCC patients (*p* < 0.05). *In vitro* studies revealed that downregulation of SREBP-1 inhibited cell proliferation and induced apoptosis in both HepG2 and MHCC97L cells (*p* < 0.05). Furthermore, wound healing and transwell assays showed that SREBP-1 knockdown prominently inhibited cell migration and invasion in both HepG2 and MHCC97L cells (*p* < 0.05). These results suggest that SREBP-1 may serve as a prognostic marker in HCC and may promote tumor progression by promoting cell growth and metastasis.

## 1. Introduction

Hepatocellular carcinoma (HCC) is the fifth most common cancer worldwide, with about 750,000 patients newly diagnosed each year [1]. As a deadly cancer, HCC is a global health problem [2]. In Asian countries, especially China, hepatitis B and C virus infection are the main risk factors for HCC development [3]. Although various treatments including hepatectomy and liver transplantation are applied in clinical practice, the prognosis for HCC patients is still not ideal. Therefore, discovering more biomarkers for HCC diagnosis and treatment is necessary.

Recent studies showed that nonalcoholic fatty liver disease (NAFLD), especially nonalcoholic steatohepatitis (NASH), is associated with an increased risk of HCC [4]. These reports suggest that abnormal lipid metabolism may play an essential role in the oncogenesis of HCC. The sterol regulatory element binding proteins (SREBPs) were originally identified in nuclear extracts in Hela cells. SREBPs are encoded by two distinct genes, SREBP-1 and SREBP-2. The SREBP-1 protein includes two different isoforms: SREBP-1a, which is mainly expressed in cultured cell lines, and SREBP-1c, which is mostly expressed in animal organs [5].

SREBP-1 functions as a transcription factor that plays a crucial role in the regulation of cholesterol, fatty acids and phospholipids synthesis [6]. Aberrant high expression of SREBP-1 has been detected in several metabolic diseases, such as diabetes mellitus, NAFLD/NASH, morbid obesity, hyperlipidemia and atherosclerosis [7]. A recent study found that silencing of SREBP-1 led to significant changes in carbohydrate metabolism via upregulation of gluconeogenesis gene expression, and decreased glycolysis and glycogen synthesis gene expression in an animal model of obesity and type 2 diabetes [8]. Additionally, SREBEP-1 overexpression has been reported in various human cancers and its abnormal expression is associated with malignant characteristics [9–13]. SREBP-1 was significantly higher in ovarian cancer compared with benign and borderline ovarian tumors. Knockdown of SREBP-1 could inhibit cell growth, migration and invasion and induce cell apoptosis in an ovarian cancer cell line [9]. In bladder cancer, SREBP-1 promotes tumor growth via regulating the expression of key lipogenic enzymes [10]. This phenomenon was also observed in endometrial cancer, prostate cancer and gastric cancer [11–13]. Despite the few studies that have shown that SREBP-1 could enhance lipid synthesis in HCC cells, the clinical significance of SREBP-1 in HCC and its role in tumor progression are still poorly understood.

In the present study, we demonstrated that positive expression of SREBP-1 was correlated with poor clinicopathological parameters, and showed that SREBP-1 was an independent prognostic factor in HCC. SREBP-1 promoted cell growth and metastasis *in vitro*. Our results suggest that SREBP-1 may promote tumor progression by promoting cell growth and metastasis in HCC.

## 2. Results

### 2.1. Clinical Significance of Elevated SREBP-1 Expression in HCC Tissues

**Figure 1.**
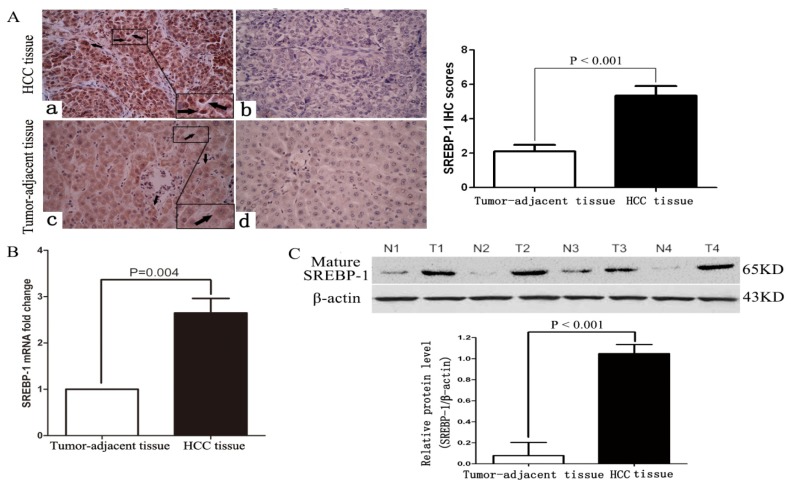
SREBP-1 expression in HCC cases. (**A**) Immunostaining of SREBP-1 in HCC and tumor-adjacent tissues: (**a**) positive expression of SREBP-1 in HCC tissue (labeled by black arrows); (**b**) negative expression of SREBP-1 in HCC tissues; (**c**) positive expression of SREBP-1 in tumor-adjacent tissues (labeled by black arrows); (**d**) negative expression of SREBP-1 in tumor-adjacent tissues (original magnification ×400). *n* = 47. Values are depicted as mean ± SD; (**B**) Expression of SREBP-1 mRNA in HCC (T) and tumor-adjacent tissues (N). *n* = 47. Values are depicted as mean ± SD; (**C**) Expression of SREBP-1 protein in HCC and tumor-adjacent tissues. *n* = 4. Values are depicted as mean ± SD.

To determine the expression of SREBP-1 in HCC tissues, we evaluated SREBP-1 mRNA and protein expression in HCC and matched normal tumor-adjacent tissues by qRT-PCR, immunohistochemistry and western blot. As shown in Figure 1A,B, both SREBP-1 mRNA and protein levels were significantly upregulated in HCC tissues compared with matched normal tumor-adjacent tissues (*p =* 0.004 and *p* < 0.001, respectively). Immunohistochemical staining of SREBP-1 could be detected in both cytoplasm and nucleus of HCC tissues. The immunohistochemistry results were further confirmed by western blot (*p* < 0.001, Figure 1C).

Clinical association analysis by the Pearson chi-square test revealed that elevated SREBP-1 expression in HCC tissues was significantly associated with large tumor size (≥5 cm; *p* = 0.005, *r* = 0.413), high histological grade (Edmondson-Steiner grade III + IV; *p* = 0.006, *r* = 0.400) and advanced tumor stage (tumor-node-metastasis (TNM) stage III + IV; *p* = 0.010, *r* = 0.378) (Table 1). These results indicate that the expression of SREBP-1 in HCC is abnormal, and that elevated SREBP-1 expression is correlated with poor clinicopathological features in HCC.

### 2.2. Positive Expression of SREBP-1 Correlates with a Worse 3-Year Survival of HCC Patients

**Figure 2.**
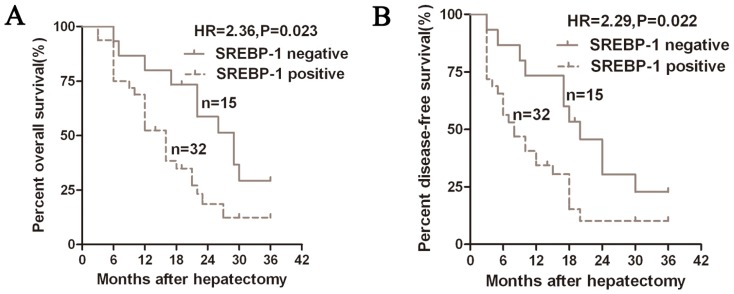
Kaplan-Meier 3-year overall (**A**) and disease-free (**B**) survival curves of HCC patients according to the status of SREBP-1 protein expression. The SREBP-1 positive expression group (*n* = 32), IHC score of SREBP-1 ≥ 1; SREBP-1 negative expression group (*n* = 15), IHC score of SREBP-1 = 0.

We next investigated whether the status of SREBP-1 expression could predict the prognosis of HCC patients. By analyzing the overall survival (OS) and disease-free survival (DFS) time of the 47 cases for the 3-year follow-up, we constructed Kaplan-Meier survival curves using overall 3-year patient survival data to analyze cases with positive and negative SREBP-1 expression. A significant correlation was detected between positive expression of SREBP-1 with shorter OS and DFS (*p* = 0.023 and *p* = 0.022, respectively, Figure 2). The OS and DFS median survival time in the SREBP-1 positive expression group were shorter than those in the SREBP-1 negative expression group (16 *vs.* 29 months and 8 *vs.* 20 months, respectively). Multivariate analysis that enrolled all of the significant clinical factors for OS and DFS indicated that SREBP-1 positive expression (*p* = 0.030 and *p* = 0.029, respectively, Table 2) is an independent prognostic factor for HCC patients. Thus, SREBP-1 may be a potential biomarker for predicting prognosis in HCC.

### 2.3. SREBP-1 Knockdown Inhibits Cell Proliferation and Induces Apoptosis in HepG2 and MHCC97L Cells

**Figure 3.**
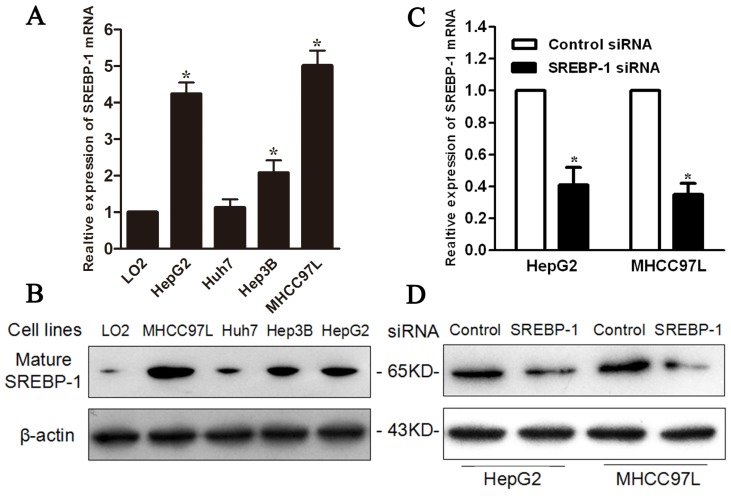
SREBP-1 is knocked down by a specific siRNA in HepG2 and MHCC97L cells. (**A**) The level of SREBP-1 mRNA in five cell lines. *n* = 3. Values are depicted as mean ± SD; * *p* < 0.05; (**B**) The level of SREBP-1 protein in five cell lines. Data are representative of multiple repeats with similar results; (**C**) SREBP-1 siRNA effectively targets SREBP-1 in HepG2 and MHCC97L cells. *n* = 3; values are depicted as mean ± SD; * *p* < 0.05; (**D**) SREBP-1 siRNA effectively decreases SREBP-1 protein in HepG2 and MHCC97L cells. Data are representative of multiple repeats with similar results.

**Figure 4.**
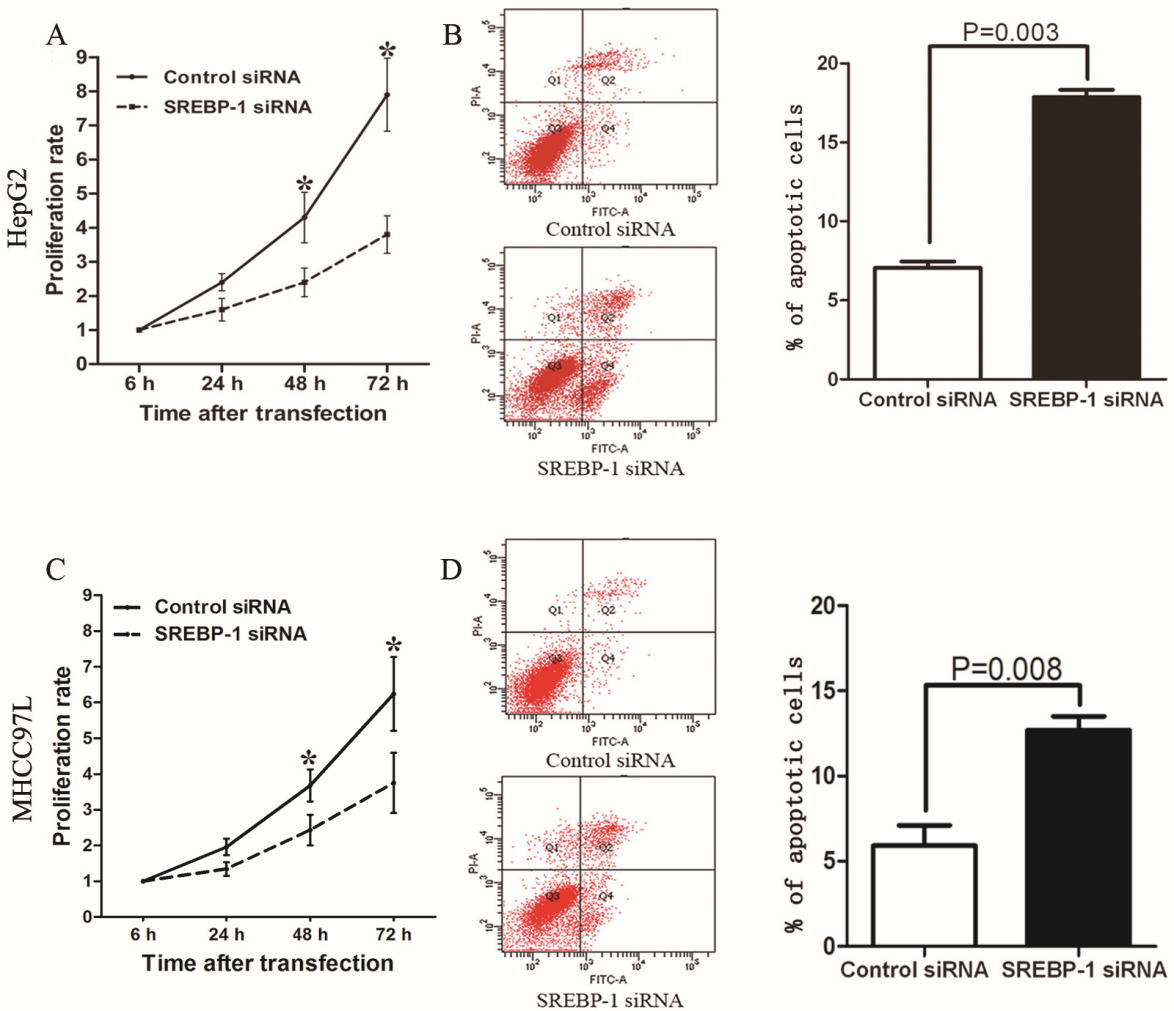
SREBP-1 regulates proliferation and apoptosis in HepG2 and MHCC97L cells. MTT assays showed that SREBP-1 knockdown reduced the viability of (**A**) HepG2 and (**C**) MHCC97L cells. *n* = 3. Data show representative results of repeat experiments. Values are depicted as mean ± SD; (**B**) and (**D**) Quantification of apoptotic cell population by flow cytometry; SREBP-1 knockdown in (**B**) HepG2 and (**D**) MHCC97L cells exhibited a larger subset of apoptotic cells compared with control cells. *n* = 3. Data show representative results of repeat experiments. Values are depicted as mean ± SD. * *p* < 0.05.

To investigate the effect of SREBP-1 in HCC cells, we first evaluated mRNA and protein levels in LO2, Hep3B, MHCC97L, Huh7 and HepG2 cells, and found both mRNA and protein were highly expressed in HepG2 and MHCC97L cells compared with the other three cell lines (Figure 3A,B). Next, we transfected specific siRNA to knockdown SREBP-1 in HepG2 and MHCC97L cells. qRT-PCR and western blot results confirmed that SREBP-1 mRNA and protein level were significantly downregulated by SREBP-1 siRNA in these two cell lines (Figure 3C,D). MTT (3-(4,5-dimethylthiazol-2-yl) 2,5-diphenyl tetrazolium bromide) assay revealed that SREBP-1 knockdown significantly inhibited HepG2 and MHCC97L cell proliferation (Figure 4A,C). Furthermore, flow cytometry demonstrated that the percentage of apoptotic cells, including both early apoptotic cells and late apoptotic cells, increased to more than two-fold after SREBP-1 knockdown in both cell lines (17.86 ± 0.47 *vs.* 7.06 ± 0.39, *p* = 0.003; 12.74 ± 0.81 *vs.* 5.91 ± 1.20, *p* = 0.008, respectively, Figure 4B,D). These results reveal that SREBP-1 may function as an oncogene by promoting cell proliferation and inhibiting apoptosis in HCC.

### 2.4. SREBP-1 Knockdown Suppresses Cell Migration and Invasion in HepG2 and MHCC97L Cells

**Figure 5.**
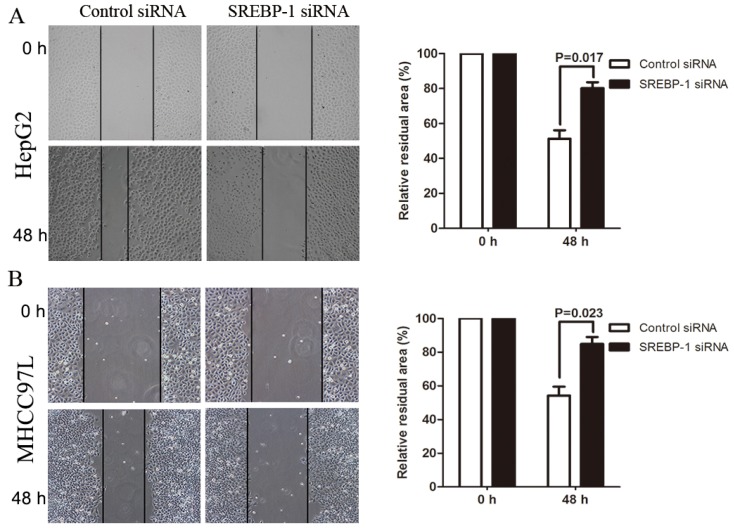
SREBP-1 regulates migration of HepG2 and MHCC97L cells. Wound healing assays showed that SREBP-1 knockdown reduced the migration of (**A**) HepG2 cells and (**B**) MHCC97L cells (original magnification ×100). *n* = 3. Data show representative results of repeat experiments. Values are depicted as mean ± SD.

**Figure 6.**
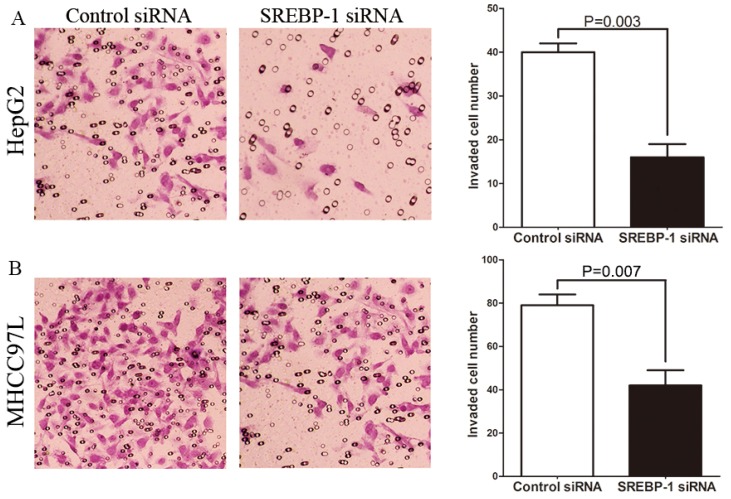
SREBP-1 regulates invasion of HepG2 and MHCC97L cells. (**A**) SREBP-1 knockdown decreased the number of invaded (**A**) HepG2 and (**B**) MHCC97L cells compared with control cells (original magnification ×200). *n* = 3. Data show representative results of repeat experiments. Values are depicted as mean ± SD.

To determine whether SREBP-1 knockdown suppresses HCC cell migration and invasion, we established mechanical scrape wound healing and transwell models. In the wound healing assay, the relative residual area was larger in the SREBP-1 siRNA group than the control group for both HepG2 and MHCC97L cells (79.59 ± 4.78 *vs.* 48.46 ± 3.57, *p* = 0.017; 84.92 ± 4.11 *vs.* 54.25 ± 5.36, *p* = 0.023, respectively, Figure 5A,B. Additionally, the number of invaded HepG2 and MHCC97L cells in the SREBP-1 siRNA group was significantly less than the control group (16.25 ± 2.17 *vs.* 41.23 ± 2.40, *p* = 0.003, 42.35 ± 7.03 *vs.* 79.71 ± 5.42, *p* = 0.007, respectively, Figure 6A,B. Together this data suggests that SREBP-1 may promote tumor metastasis in HCC.

## 3. Discussion

The maintenance of intracellular lipid homeostasis relies on the balance between lipid biosynthesis and decomposition. Several studies have demonstrated that aberrant expression of lipogenic enzyme genes generally occurs in liver cancer cells and contributes to tumor progression. Aberrant expression is mainly caused by inappropriate transcriptional activation via several transcription factors, such as SREBP-1, SREBP-2 and HNF-4α [14–16]. Among these transcriptional control factors, SREBP-1 was considered as the most important regulator in HCC.

Our previous study revealed that the expression of SREBP-1 in the liver is markedly increased in a mouse model of NAFLD [17]. SREBP-1 may be correlated with HCC from NAFLD. In this study, we demonstrated that SREBP-1 was significantly upregulated in HCC tissues compared with normal tumor-adjacent tissues, and elevated SREBP-1 expression was significantly associated with large tumor size (>5 cm), high Edmonson-Steiner classification (stage III + IV) and advanced TNM stage (stage III + IV). Kaplan-Meier analysis revealed that SREBP-1 positive expression was correlated with a poorer survival of HCC patients after hepatectomy. Additionally, Yamashita *et al.* also confirmed that SREBP-1 positive expression was associated with high mortality in HCC patients [18]. Furthermore, multivariate Cox repression analysis found that SREBP-1 was an independent factor for predicting both 3-year overall and disease-free survival in HCC patients. These results show that the status of SREBP-1 is critical for prognosis determination in HCC patients.

Our *in vitro* studies found that SREBP-1 knockdown by a specific siRNA inhibited cell proliferation and induced apoptosis in both HepG2 and MHCC97L cells, which was consistent with previous studies [18]. Cell migration and invasion are the cytological basis in tumor metastasis. Our results demonstrated for the first time that SREBP-1 knockdown suppressed cell migration and invasion in HepG2 and MHCC97L cells. SREBP-1 has been found to directly promote the gene transcription of fatty acid synthase (FASN), acetyl-CoA carboxylase (ACAC), and 3-hydroxy-3-methylglutaryl-coenzyme A reductase (HMGCR) by promoter analysis [19,20]. FASN overexpression is associated with resistance to several chemotherapeutic drugs, such as paclitaxel, vinblastine, doxorubicin and methotrexate, in HCC, and prevents HCC cells from apoptosis induced by these drugs [21]. Suppression of the genes *ACAC*, *FASN*, or *SREBP-1*, which are involved in lipid metabolism, decreases lipogenesis and reduces proliferation and survival of HCC cells through an AKT-mTORC1-RPS6 pathway [22]. HMGCR was shown to be overexpressed and led to cholesterol overproduction in HCC tissues [23]. HMGCR regulates the phosphorylation status of MYC, which is known as a crucial oncogene in HCC, and affects tumor growth *in vitro* and *in vivo* [23]. Additionally, all these enzymes also participate in cell migration and invasion in human glioma, osteosarcoma and prostate cancers [24–27]. Therefore, SREBP-1 may play its carcinogenic role through these molecular signaling pathways.

## 4. Experimental Section

### 4.1. Ethical Review

All protocols were approved by the Xi’an Jiaotong University Ethics Committee according to the 1975 Declaration of Helsinki. Informed consent was obtained and signed by each patient.

### 4.2. Clinical Samples and Cell Lines

A total of 47 HCC patients including 29 males and 18 females (range, 36–73 years; median 51 years) who underwent curative liver resection at Department of Hepatobiliary Surgery, First Affiliated Hospital of Medical College of Xi’an Jiaotong University during March 2010 to November 2010 were included in this study. Patients had a median follow-up time of 22.5 months. All patients did not receive any chemotherapy, radiotherapy or radiofrequency ablation before operation. HCC tissues and matched normal tumor-adjacent tissues(>2 cm distance of the surgical margin) were collected and immediately stored in 4% paraformaldehyde solution for immunohistochemistry (IHC) or liquid nitrogen for western blot. The demographic and clinicopathological characteristics data were obtained from the medical records.

The human immortal liver cell line LO2 and four HCC cell lines, Hep3B, MHCC97L, Huh7, HepG2, were obtained from the Institute of Biochemistry and Cell Biology, Chinese Academy of Sciences (Shanghai, China). Cells were cultured in complete Dulbecco’s modified Eagle medium (DMEM, Mediatech, Manassas, VA, USA) containing 10% fetal bovine serum (FBS, Gibco BRL, Gaithersburg, MD, USA) in a humidified 5% CO_2_ incubator at 37 °C. The logarithmic growth phase cells were harvested for further assays.

### 4.3. Immunohistochemical Analysis

Immunohistochemistry was performed on paraformaldehyde-fixed paraffin sections. SREBP-1 (2A4, LabVision&Neomarkers, Waltham, MA, USA) (1:100) antibodies were used in immunohistochemistry with the streptavidin peroxidase-conjugated (SP-IHC) method. Immunohistochemistry was performed as previously reported [2]. The staining results for the SREBP-1 protein were semiquantitatively calculated by multiplying the staining intensity and the percentage of positive liver cells. Staining intensity was expressed as four grades: 0 = none; 1 = weak; 2 = moderate; and 3 = strong. The percentage of positive liver cells was expressed as the following grades: 0, <5%; 1, 6%–25%; 2, 26%–50%; 3, 51%–75%; and 4, >75%. Ten independent high magnification (×400) fields were assayed for each section, and the average scores of ten fields determined the final results. Sections with a total score of more than 1 were defined as exhibiting positive staining for the SREBP-1 protein.

### 4.4. siRNA Transfection

The specific siRNA against SREBP-1 (predesigned siRNA, Table 3) and scramble siRNA were synthesized by GenePharma Co., Ltd. (Shanghai, China). HepG2 cells were seeded in 6-well plates at the concentration of 2 × 10^5^/well, and divided into two groups: the SREBP-1 siRNA group and the control siRNA group. HepG2 or MHCC97L cells were transfected with 150 pmol siRNAs (SREBP-1 siRNA or control siRNA) using 5 μL Lipofectamine 2000 (Invitrogen, Carlsbad, CA, USA) according to the manufacturer’s instructions, and cultured in a humidified 5% CO_2_ incubator at 37 °C. Reduced serum medium was changed to complete medium 6 h after transfection. Cells were harvested 48 h after transfection and used for further experiments.

### 4.5. Quantitative Real-Time Reverse Transcription Polymerase Chain Reaction (qRT-PCR)

Total RNA was isolated from HCC tissues and HCC cells using TRIZOL^®^ reagent (Invitrogen, Carlsbad, CA, USA) according to the manufacturer’s instructions. The first strand cDNA was synthesized using the Revertid™ First Strand cDNA Synthesis Kit (Fermentas, Burlington, ON, Canada). cDNA (2 μL) obtained from each sample was amplified and quantified by real-time PCR using SYBR^®^ Premix Ex TaqTM II (Tli RNaseH Plus, Takara, Shiga, Japan). The human GAPDH gene served as an internal control gene to ensure that an equal amount of mRNA was analyzed from each sample. The primers were synthesized by GenePharma Co., Ltd. (Shanghai, China) and the sequences are shown in Table 3. Relative gene expression was calculated with the 2^−ΔΔ^*^C^*^t^ method. Three independent experimental replicates were performed.

### 4.6. Immunoblot Analysis

SREBP-1 (1:1000) and β-actin (Santa Cruz, Dallas, TX, USA) (1:5000) antibodies were used for western blot assay. Secondary horseradish peroxidase-conjugated goat anti-mouse antibody (Bio-Rad, Hercules, CA, USA) were used at a 1:5000 dilution and detected by the Enhanced Chemiluminescence Reagent (Millipore, Billerica, MA, USA).

### 4.7. MTT Assay

Proliferation viability was determined using the 3-(4,5-dimethylthiazol-2-yl)-2,5-diphenyltetrazolium bromide (MTT, Roche Diagnostics, Mannheim, Germany) assay. Cell viability was calculated at 24, 48 and 72 h after transfection. The absorbance of the samples was measured using a model 550 microplate reader (Bio-Rad Laboratories, Hercules, CA, USA) at a wavelength of 490 nm. Three independent experimental replicates were performed.

### 4.8. Flow Cytometry

Cell apoptosis were analyzed by the Annexin-V-FLUOS Staining Kit (Roche Diagnostics, Mannheim, Germany) 48 h after transfection. The samples were analyzed with a BD FACS Canto II Flow Cytometer (Becton Dickinson, Franklin Lakes, NJ, USA). Three independent experimental replicates were performed.

### 4.9. Wound Healing Assay

The cells were seeded in 6-well plates at a high density and allowed to form cell monolayers overnight. A 200 μL sterile plastic tip was used to create a wound line across the surface of plates, and the suspension cells were removed with PBS. Cells were cultured in reduced serum DMEM medium in a humidified 5% CO_2_ incubator at 37 °C for 48 h, and then images were taken with a phase-contrast microscope.

### 4.10. Transwell Assay

The 8 μM pore-sized transwell inserts (Nalge Nunc, Penfield, New York, NY, USA) were coated with matrigel (BD Biosciences, Franklin Lakes, NJ, USA) at 1 mg/mL on the inner layer. HepG2 cells were resuspended with reduced serum DMEM medium and the density was adjusted to 2.5 × 10^5^/mL 48 h after transfection. A 200 μL cell suspension was added into the upper chamber, and 750 μL DMEM medium containing 10% FBS was added into the lower chamber, and then incubated for 24 h. Cells were fixed in 4% paraformaldehyde for 2 min, and then permeabilized in methanol for 20 min. The cells on the inner layer were softly removed with a cotton swab, and the adherent cells on undersurface of the insert were stained with 0.3% crystal violet dye for 15 min. The filters were washed with PBS and images were taken. Invaded cells on undersurface were counted under a light microscope.

### 4.11. Statistical Analysis

All data are presented as mean ± SD. The SPSS statistical package for Window Version 13 (SPSS, Chicago, IL, USA) was used for Pearson chi-square test and the multivariate Cox regression analysis. Two-tailed Student’s *t* test, Kaplan-Meier plots and log-rank test or ANOVA was used to evaluate statistical significant using GraphPad Prism 5 software (GraphPad Software, Inc., San Diego, CA, USA). *p* < 0.05 was considered statistically significant.

## 5. Conclusions

In conclusion, this study demonstrates that SREBP-1 is upregulated in HCC tissues and its overexpression is correlated with poor clinicopathological features. Furthermore, we showed that SREBP-1 positive expression is associated with shorter overall and disease-free survival time of HCC patients after hepatectomy. Multivariate Cox repression analysis indicates SREBP-1 as an independent risk factor for predicting the prognosis of HCC patients after operation. We also showed that SREBP-1 knockdown inhibits cell proliferation, migration and invasion, and induces apoptosis in both HepG2 and MHCC97L cells, suggesting that SREBP-1 may promote tumor progression by promoting cell growth and metastasis. Together, our findings suggest that SREBP-1 could be a potential therapeutic target for HCC.

## Figures and Tables

**Table 1.** Clinical correlation of SREBP-1 expression in HCC (*n* = 47).

Clinicopathologic	Features	Total No. of Patients, *n* = 47	No. of Patients		*p*	*r*

			SREBP-1 Negative	SREBP-1 Positive		
Age (y)	<50	13	3	11	0.508	–0.147
	≥50	34	12	21		
Sex	Male	30	11	19	0.353	0.135
	Female	17	4	13		
HBV	Absent	8	3	5	0.710	0.054
	Present	39	12	27		
Serum AFP level (ng/mL)	<400	22	8	14	0.539	0.090
	≥400	25	7	18		
Tumor size (cm)	<5	15	9	6	0.005 *	0.413
	≥5	32	6	26		
Cirrhosis	Absent	9	5	4	0.196	0.247
	Present	38	10	28		
Venous infiltration	Absent	25	11	14	0.058	0.276
	Present	22	4	18		
Number of nodules	1	27	11	16	0.123	0.220
	≥2	20	4	16		
Edmondson-Steiner grading	I + II	18	10	8	0.006 *	0.400
	III + IV	29	5	24		
TNM tumor stage	I + II	28	13	15	0.010 *	0.378
	III + IV	19	2	17		

HCC, hepatocellular carcinoma; HBV, hepatitis B virus; AFP, alpha-fetoprotein; TNM, tumor-node-metastasis;

* Statistically significant.

**Table 2.** Multivariate Cox regression analysis of 3-year OS and DFS of 47 HCC patients.

Variables	Overall Survival			Disease-Free Survival		
	
	HR	95% CI	*p*	HR	95% CI	*p*
Edmondson-Steiner grade	1.695	0.784–3.663	0.043 *	1.646	0.579–4.680	0.035 *
TNM stage	4.207	1.547–11.437	0.005 *	7.363	2.134–25.400	0.002 *
SREBP-1 expression in HCC	2.976	1.109–7.987	0.030 *	2.327	1.093–4.955	0.029 *

HR, hazard ratio; CI, confidence interval;

* Statistically significant.

**Table 3.** Primers for real-time PCR and SREBP-1 siRNA sequence.

Primers and siRNA	5′-3′	
SREBP-1 primer	F	CAGTCCAGCCTTTGAGGATA
	R	CAAAGGATTGCAGGTCAGAC
GAPDH primer	F	CAAGCTCATTTCCTGGTATGAC
	R	CAGTGAGGGTCTCTCTCTTCCT
SREBP-1 siRNA	Sense	GGAAGAGUCAGUGCCACUGTT
	Anti-sense	CAGUGGCACUGACUCUUCCTT
